# Nursing as the profession of the future and the foundation of universal health systems

**DOI:** 10.1590/1518-8345.0000.3063

**Published:** 2018-10-11

**Authors:** Ricardo Alexandre Arcêncio

**Affiliations:** 1 Universidade de São Paulo, Escola de Enfermagem de Ribeirão Preto, PAHO/WHO Collaborating Centre for Nursing Research Development, Ribeirão Preto, SP, Brazil.

**Figure:**
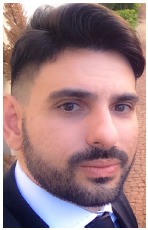

A serious crisis has engulfed Brazil in recent years, threatening the access to the universal health system and setting back the achievements of the last decades. This editorial aims to present relevant elements, necessary for the sustainability of a universal system (universal system^1^ must not be understood as universal coverage system), with a focus on nursing, which is the basis for a project with breadth and social value.

Brazil is one of the only large countries (today with a population of just over 209 million people) that has adopted the universal system. The system operates with more than 3.5 million workers, of which about 50% work in nursing. This category is present in all Brazilian cities, with even greater prevalence in the public, philanthropic and private sectors^2^.

In spite of the considerable number of nursing professionals (6.15 professionals per 1,000 inhabitants in 2015), it still does not meet the Brazilian social needs and falls below countries such as Canada (9.84), Sweden (11.87), United Kingdom (8.43) and Uruguay (12.49)^3^. In Brazil, the number of nurses is much lower than the number of nursing technicians and assistants - a reality consistent with global estimates, which predict that the world will need an additional 9 million nurses and midwives to attend to the health needs of the planet by 2030.

This context motivated the World Health Organization (WHO) and the United Nations (UN) to launch the Nursing Now Program^1^, which has the Duchess of Cambridge, Kate Middleton, as its staunch advocate and patron of the initiative. Its main objectives is eliminating the shortfall of professionals, stimulating the creation of programs to improve incentive, visibility, valorization and employment conditions of the nursing professional.

On the situation of the nursing profession in Brazil, based on data collected from DATASUS^2^, there are imbalances between nurses and nursing technicians growing trends in the Unified Health System (SUS) and in the private sector. Between 2006 and 2016, the number of nurses hired for SUS has grown in only ten Brazilian States, while in most of them the situation remains unchanged; as for nursing technicians and/or assistants hired for the SUS, their numbers have increased in all states. It is important to emphasize that the increase in the number of nurses is much higher in the private system than in the public system and that there is a smaller increase of technicians in this sector, which has important implications for the universal system.

The reality of Brazilian nursing is a reflection of recent policies of austerity, cuts, containment of public expenditures, provisional measures that allow foreign capital flows, and the Política Nacional da Atenção Básica itself, which recognizes and finances other modalities of attention other than the Family Health Strategy. In addition, the private system and the market economy have repercussions on low salaries, long hours of work of the nurse (with delays in the approval of Law 2295/00, for 30 working hours/week) and occupational stress.

We emphasize that, for the sustainability of the universal system, the nurse must be one of its main allies, since this professional is the one that has most incorporated the practice of caring. The values, vision and mission of this profession are intertwined with the exercise of care, which means giving attention, treating, respecting and attending the human being in all their needs.

Several countries have movements to define the competencies expected for nurses, considering social determinants and inequities (which have reached critical levels in recent years with civil/religious wars, immigration, austerity policies, among others), aging (there will be nearly 2 billion elderly people by 2050) and an increasingly demanding, distrustful and unsatisfied population.

The advanced nursing practice is a relevant mechanism for supporting the leading role of nurses in health production in Latin America and the Caribbean. However, there are other references and even complementary ones that not only address the increase of the actions covered by the nurse in quantitative terms, but redesign the profession in the qualitative aspects, defining the essential core of the profession and its horizons in the contemporary world, mediated by equity, integrity, justice, law and ethics.

Thus we bring the *Nurse of the future nursing core competencies,* produced by the Massachusetts Department of Higher Education Nursing Initiative, which emphasizes the art and science of care^4^. It defines ten essential nurses’ competencies for the present and for the future, which include patient-centered care, professionalism, leadership and strategic management, recording technologies and computer skills, evidence-based practice, communication (including social participation), systems and processes based on best practice, safety, teamwork, cooperation/coordination of care and quality improvement.

These guidelines have influenced the training and practice of nurses in the United States of America and today serve as an interesting basis for thinking about nurses’ curricula and practice in universal systems. Evidently considering the economic, political, ideological and cultural differences and the distinctions between the logics of organization of the health system in our country and in the US (free market), we find in this reference the perspective of a bold nursing, with the breadth and perspective expected.

We do not intend to close with a single reference, but to open up new possibilities that consider the nurse as a necessary professional now and in the future, support political decisions, guaranteed by the Welfare State and human dignity.

It is imperative and strategic to think about nursing today. Based on these reflections we can find forms of health production consistent with the fundamental theoretical-philosophical, legal and ethical basis of health systems. Among the professions, it is the one that most assimilates the values of universal systems. Therefore political commitment, along with critical mass and social capital, are necessary to defend this proposal and this profession.

## Footnotes

1 http://www.nursingnow.org

## Footnotes

2 http://datasus.saude.gov.br/
